# New-Onset Heart Failure in the Setting of T4-Conversion Disorder

**DOI:** 10.7759/cureus.25024

**Published:** 2022-05-15

**Authors:** Nicole Chan, Kevin Pak, Alan Guo, Pranav Singla, Mark Sayegh

**Affiliations:** 1 Internal Medicine, St. John's Riverside Hospital, Yonkers, USA; 2 Internal Medicine, Lake Erie College of Osteopathic Medicine, Eerie, USA; 3 Neurobiology and Behavior, Stony Brook University, Stony Brook, USA

**Keywords:** deiodinase, heart failure with reduced ejection fraction, liothyronine, levo-thyroxine, hypothyroid

## Abstract

Thyroid hormone is essential in accomplishing the appropriate metabolism of the body. Achieving euthyroidism is of importance due to the deadly ramifications of being hypothyroid, such as multiple organ failure, profound decrease in mentation and even death.

We present a case of an 80-year-old female with a history of hypertension, coronary artery disease, chronic kidney disease, hypothyroidism due to total thyroidectomy, and a cerebral vascular accident who presented with slurred speech, decreased appetite, dizziness and lethargy with new-onset weakness. She was adherent to all her medications. Her labs were significant for elevated thyroid-stimulating hormone, elevated free thyroxine, and low total triiodothyronine. Brain MRI revealed no acute pathology. She was given her home dose of Levothyroxine and was admitted to the telemetry unit for evaluation of her symptoms and abnormal thyroid panel. During her hospital course, she was found to have an abnormal rhythm and worsening lethargy. Subsequent electrocardiogram and laboratory values revealed new T-wave inversions and elevated troponin. An echocardiogram revealed a new severely reduced left ventricular function with severe global hypokinesis of the left ventricle and an ejection fraction of 30%. It was only after initiating combination therapy of levothyroxine and liothyronine that her symptoms and abnormal cardiac rhythm resolved. With this careful titration of the patient’s medication, we concluded that combination therapy was essential to the patient being euthyroid. This phenomenon was also cited in multiple literature, which warrants an investigation of a certain population’s inability to convert T4 to T3.

By sharing this case, we aim to aid providers with their differential diagnoses and bring to light a potential area of further investigation. Ultimately, by optimizing and tailoring these medications, we hope to improve their quality of life.

## Introduction

Hypothyroidism is a disorder consisting of insufficient amounts of thyroid hormone to sustain the metabolism of the body. Nearly 10 million people (4.6%) in the United States have hypothyroidism and studies have shown that those who are given thyroid hormone therapy have associated reverse myocyte apoptosis, improved cardiovascular performance, decreased total cholesterol, low-density lipoproteins (LDLs) and triglycerides [1,2]. Symptoms of hypothyroidism include fatigue, poor concentrating abilities, weight gain, poor appetite, constipation, and cold intolerance. To supplement the thyroid gland, providers have been using exogenous levothyroxine in order to assist in the homeostatic processes necessary to sustain life. We present a case in which an individual is minimally responsive to levothyroxine and developed new-onset heart failure despite being adherent to her medication due to a T4-conversion disorder. This highlights the importance of routine follow-up in regards to patients who are on thyroid hormone therapy, as they may need supplemental agents to achieve euthyroidism and reduce mortality associated with uncontrolled hypothyroidism.

## Case presentation

This is an 80-year-old African American female with a history of hypertension, coronary artery disease, chronic kidney disease, hypothyroidism due to total thyroidectomy, and a cerebral vascular accident with residual left-sided deficit who presented to the emergency department with two episodes of slurred speech (one day and six hours prior to presenting to the hospital) and two days of decreased appetite, dizziness and lethargy. She was also noted to have new-onset weakness and required assistance ambulating to the bathroom. At baseline, she is usually able to ambulate independently with a walker. There were no recent changes in medications. Vitals on admission were the following: Blood pressure 131/63, heart rate 71, temperature 97.9. Physical exam was significant for her being alert and oriented to only herself (at baseline, she is oriented to self and place), left facial nasolabial flattening, decreased left side facial sensation, and decreased muscle strength in bilateral upper and lower extremities, right side being worse than her left. There was no notable swelling in her legs. Unfortunately, cortisol level was not evaluated at this time. Her labs were significant for macrocytic anemia, elevated blood urea nitrogen and creatinine (measured at 2.9 mg/dL with her baseline creatinine being 1.5 mg/dL), elevated cholesterol (467), elevated LDL (357), elevated thyroid-stimulating hormone (29.1), elevated free thyroxine (1.59), and low total triiodothyronine (T3) (<20). The previous measurement of her TSH was 1.76, which is within normal limits. Notably, a year prior to presentation, the patient had suffered from a transient ischemic attack and TSH measured at the time was 0.01 (below normal). Her thyroid medication was adjusted during this acute illness, but she resumed her usual dose upon discharge and continued the dose without issue until presentation (Table 1).

**Table 1:** Thyroid panel trends during hospitalization TSH: thyroid-stimulating hormone, T4: thyroxine, T3: triiodothyronine

Lab name (Reference range and units)	On Day of Admission	Day 6	Day 11
TSH (0.358– 3.74 μLU/mL)	29.10	29.60	11.50
Free T4 (0.76 – 1.46 ng/dL)	1.59	1.52	1.43
Total T3 (71 – 180 ng/dL)	<20.00	39.00	79.00

She had a CT head without contrast, which revealed no evidence of acute pathology. Subsequent brain MRI revealed only moderate atrophy and periventricular microvascular ischemic changes (Video 1). The patient’s family members who have been caring for her have been ensuring that the patient had been taking her prescribed medications routinely and correctly. She was given her home dose Levothyroxine 150mcg in the Emergency Department and admission was requested for lethargy and weakness in the setting of her stroke history. Given negative brain MRI, the patient’s lethargy, weakness, and decreased appetite these symptoms are likely due to her hypothyroidism.

**Video 1:** CT head and Brain MRI

**Figure 1:**
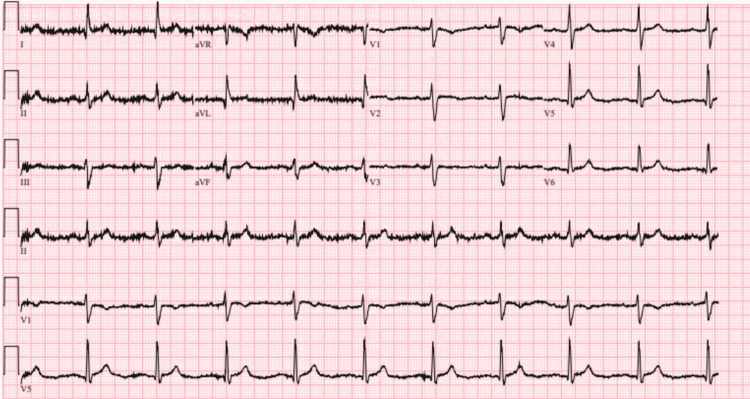
Electrocardiogram on admission

**Figure 2:**
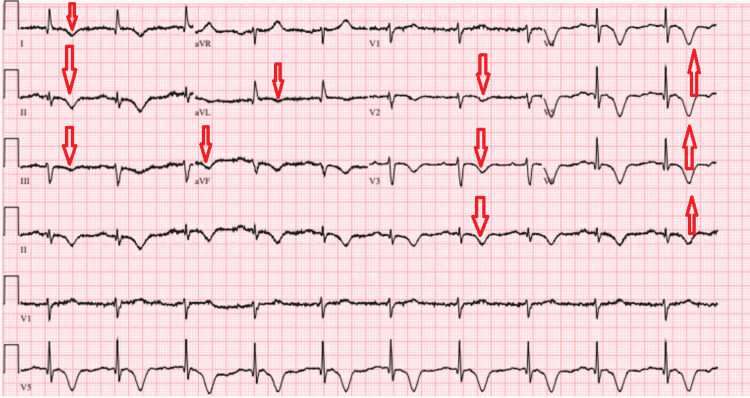
Follow-up electrocardiogram with diffuse T-wave inversions

She was admitted to the telemetry unit, where she was found to have an abnormal rhythm and worsening lethargy. Electrocardiogram revealed normal sinus rhythm and diffuse nonspecific T-wave inversions (Figures 1, 2). Laboratory drawn at that instance revealed elevated troponin of 0.53, which trended down to 0.25. It was noted that an echocardiogram done the previous year was essentially unremarkable, but repeat echocardiogram revealed severely reduced left ventricular function with severe global hypokinesis of the left ventricle and an ejection fraction of 30%.

The patient was initiated on levothyroxine intravenously, but repeated thyroid panels showed thyroid stimulating hormone trending up, persistently elevated free thyroxine and low T3 despite therapy. She was given liothyronine in combination with her current therapy. Five days after this combination therapy, the patient’s weakness and lethargy improved. The thyroid-stimulating hormone decreased to near-normal values and her thyroxine and T3 returned to normal values (Table 1). She was discharged with oral levothyroxine and liothyronine, physical therapy, instruction for repeat echocardiogram and evaluation of her lipid panel and thyroid panel to ensure their return to normal values.

## Discussion

Hypothyroidism is a disease of insufficient thyroid hormone that affects all organ systems. Symptoms include cold intolerance, dry skin, constipation, decreased appetite, weakness, fatigue, delayed reflexes and bradycardia. Due to the diverse presentation of hypothyroidism and lack of specificity in its manifestation, its diagnosis is dependent on laboratory values. In our patient, the presentation of hypothyroidism occurred despite adherence to levothyroxine. It is well known that taking levothyroxine with certain oral nutrition (especially soy products, calcium, iron and dietary fiber) may cause reduced bioavailability in serum thyroxine levels [3,4]. However, the patient in this case always took her medications one hour prior to her first meal. It was noted that none of her other medications interfere with absorption of levothyroxine. Due to the erratic bioavailability of this medication and the question of adequate absorption of the medication, the patient received the intravenous form of the medication for faster onset of action, within 6-8 hours of administration. Despite this, the patient’s original presentation persisted and the values of the thyroid panel remained abnormal. Interestingly, these lab values were consistent with what AAFP literature points to as a disruption of the T4-T3 converting enzyme [5]. Thus, the limiting factor is speculated to be at the level of deiodinase enzymes, which catalyzes the activation of thyroid hormone. Ultimately, this process generates T3, the active form of thyroid hormone. A study by Fish et al. from the New England Journal of Medicine further highlights the importance of T3, in regulation of TSH. These findings indicate that even small reduction in T3 could contribute to a rise in the TSH [6]. The patient did not respond to IV levothyroxine therapy, but obtained near euthyroid levels after initiating liothyronine. This bypasses the conversion of active thyroid hormone via the deiodinase. Correction of TSH followed the improvement of T3, which suggests a defect in conversion of T4-T3.

Deiodinases are imperative in both activation of thyroxine and inactivation of T3 to maintain homeostatic function at the plasma and cellular level. Deiodinase 2 (D2) has been shown to be upregulated in hypothyroidism to elevate production of serum T3. It has been shown to contribute to as much as 50% of T3 content in some tissues. On the other hand, deiodinase 3 (D3) has been shown to inactivate T3 and contributes to the homeostatic levels of maintaining euthyroidism. A 2015 study reported elevated levels of T4 shortens D2 half-life from hours to 20 minutes in thyroidectomized rodents. This suggests that thyroid hormone regulation in levothyroxine-treated patients could decrease thyroid hormone signaling that permits D2 expression in tissues, which would ultimately lead to decreased amount of active thyroid hormone [7,8].

The patient was uncertain as to why she had undergone a total thyroidectomy. However, the fact of her having this procedure is significant because of studies regarding polymorphisms in the thyroid hormone pathway in thyroidectomized patients. The most studied genetic variant is Thr92Ala in DIO2 (a Protein Coding gene for D2). This population of patients with this polymorphism revealed decreased D2 activity and subsequent lower serum T3 after thyroidectomy compared to those of the wild-type patients. Notably, findings of this study also found that this mutant allele was less efficient than wild-type D2 in converting T4 into T3 in muscle cells in vivo [9,10]. Our patient may have had this mutant allele, which could explain why she had deficiencies in her ability to convert T4 to T3 and eventually became hypothyroid. One rare and life-threatening result of hypothyroidism in which the body can no longer compensate for is a condition called myxedema coma, which, if hypothyroidism is not adequately treated for, can precipitate to a fatal outcome.

Myxedema coma is a life-threatening form of hypothyroidism with a 40% mortality rate, which is why it is imperative for healthcare providers to recognize and treat promptly before detrimental results. One should suspect a diagnosis of myxedema coma if patients present with profound hypothermia, decreased mentation, generalized edema and other symptoms of worsening hypothyroidism, such as bradycardia, hypotension, hypoglycemia or respiratory failure requiring mechanical ventilation. This condition is most commonly seen in women who are over the age of 60 with insufficiently treated hypothyroidism and is most often precipitated by factors such as infections or cessation of thyroid medication [11,12]. This differs from hypothyroidism in that the severity of these symptoms overwhelm the body’s compensatory mechanism in maintaining physiological functions. Due to this, myxedema coma requires higher level of care via the intensive care unit with meticulous cardiac and respiratory function monitoring. Our patient did not exhibit such severe signs or symptoms; however, given her age and her being a woman, she most definitely had risk factors that would have predisposed her to developing myxedema coma.

Further research is warranted in discerning deiodinase characteristics and function in the human body. Currently there is no test to determine levels of deiodinase and its activity in humans. The ultimate challenge lies in recording deiodinases’ various locations and types located in the human body. Despite this, new testing abilities should be pursued to discern functionality of deiodinases in the human population. Our patient had new-onset heart failure and electrocardiographic changes in the setting of severe hypothyroidism. This is due to the necessity of triiodothyronine in preserving both cardiac morphology and performance in adult life. The maintenance of euthyroidism is important due to the potential negative impact of low-T3 state on the prognosis of cardiac diseases. One prospective study found that a low-T3 state was associated with higher rates of cardiac death [13,14]. In addition to the case presented here, there have been other documented cases of patients who remain symptomatic with hypothyroidism despite being on levothyroxine. A study of 1,000 patients observed for one year with hypothyroidism were placed on both levothyroxine and liothyronine therapy and subsequently achieved euthyroidism. Additionally, observations of 400 of these patients for approximately nine years did not reveal increased morbidity or mortality due to cardiovascular disease [15]. Another retrospective study of 1,811 individuals with thyroid dysgenesis revealed that there is heterogeneity in the ability for individuals to convert T4 into T3. There is a possibility that these individuals have abnormal or heterogeneity in deiodinase activity, which was addressed after giving combined therapy [16]. Thus, providers must be vigilant in monitoring patients with risk of hypothyroidism despite being on supplemental thyroid hormone therapies as they may develop T4-conversion disorder.

## Conclusions

Providers should be mindful of patients who may have T4-conversion disorder, as low T3 is a strong prognostic predictor of death in patients with heart disease. Further research is warranted in discovering the pathophysiology in which T4-conversion disorders manifest. With this, optimal thyroid hormone therapy may be tailored for these individuals and improve their quality of life. By sharing this case, we aim to aid providers with their differential diagnoses and bring to light a potential area of further investigation.

## References

[1] Tanguay M, Girard J, Scarsi C, Mautone G, Larouche R (2019). Pharmacokinetics and comparative bioavailability of a levothyroxine sodium oral solution and soft capsule. Clin Pharmacol Drug Dev.

[2] Colucci P, Yue CS, Ducharme M, Benvenga S (2013). A review of the pharmacokinetics of levothyroxine for the treatment of hypothyroidism. Eur Endocrinol.

[3] Hueston WJ (2001). Treatment of hypothyroidism. Am Family Phys.

[4] Fish LH, Schwartz HL, Cavanaugh J, Steffes MW, Bantle JP, Oppenheimer JH (1987). Replacement dose, metabolism, and bioavailability of levothyroxine in the treatment of hypothyroidism. Role of triiodothyronine in pituitary feedback in humans. N Engl J Med.

[5] Gereben B, Zeöld A, Dentice M, Salvatore D, Bianco AC (2008). Activation and inactivation of thyroid hormone by deiodinases: local action with general consequences. Cell Mol Life Sci.

[6] Werneck de Castro JP, Fonseca TL, Ueta CB (2015). Differences in hypothalamic type 2 deiodinase ubiquitination explain localized sensitivity to thyroxine. J Clin Invest.

[7] Klein I, Ojamaa K (2001). Thyroid hormone and the cardiovascular system. N Engl J Med.

[8] Francois J, Al-Sadawi M, Casillas J (2020). Hypothyroidism and heart failure: epidemiology, pathogenetic mechanisms & therapeutic rationale. Int J Clin Res Trials.

[9] Castagna MG, Dentice M, Cantara S (2017). DIO2 Thr92Ala reduces deiodinase-2 activity and serum-T3 levels in thyroid-deficient patients. J Clin Endocrinol Metab.

[10] Wiersinga WM (2017). Therapy of endocrine disease: T4 + T3 combination therapy: is there a true effect?. Eur J Endocrinol.

[11] Pangtey GS, Baruah U, Baruah MP, Bhagat S (2017). Thyroid emergencies: new insight into old problems. J Assoc Phys India.

[12] Ylli D, Klubo-Gwiezdzinska J, Wartofsky L (2019). Thyroid emergencies. Pol Arch Intern Med.

[13] Iervasi G, Pingitore A, Landi P (2003). Low-T3 syndrome: a strong prognostic predictor of death in patients with heart disease. Circulation.

[14] Clyde PW, Harari AE, Getka EJ, Shakir KM (2003). Combined levothyroxine plus liothyronine compared with levothyroxine alone in primary hypothyroidism: a randomized controlled trial. JAMA.

[15] Idrees T, Palmer S, Maciel RM, Bianco AC (2020). Liothyronine and desiccated thyroid extract in the treatment of hypothyroidism. Thyroid.

[16] Gullo D, Latina A, Frasca F, Le Moli R, Pellegriti G, Vigneri R (2011). Levothyroxine monotherapy cannot guarantee euthyroidism in all athyreotic patients. PLoS One.

